# Development of a complex intervention to test the effectiveness of peer support in type 2 diabetes

**DOI:** 10.1186/1472-6963-7-136

**Published:** 2007-08-31

**Authors:** Gillian Paul, Susan M Smith, David Whitford, Fergus O'Kelly, Tom O'Dowd

**Affiliations:** 1 Department of Public Health and Primary Care, Trinity College, Dublin 2, Ireland; 2 Department of General Practice, RCSI, Dublin 2, Ireland; 3 Trinity College-Eastern Regional General Practice Training Programme, Health Services Executive, Dublin 2, Ireland

## Abstract

**Background:**

Diabetes is a chronic illness which requires the individual to assume responsibility for their own care with the aim of maintaining glucose and blood pressure levels as close to normal as possible. Traditionally self management training for diabetes has been delivered in a didactic setting. In recent times alternatives to the traditional delivery of diabetes care have been investigated, for example, the concept of peer support which emphasises patient rather than professional domination. The aim of this paper is to describe the development of a complex intervention of peer support in type 2 diabetes for a randomised control trial in a primary care setting.

**Methods:**

The Medical Research Council (MRC) framework for the development and evaluation of complex interventions for randomised control trials (RCT) was used as a theoretical guide to designing the intervention.

The first three phases (Preclinical Phase, Phase 1, Phase 2) of this framework were examined in depth. The Preclinical Phase included a review of the literature relating to type 2 diabetes and peer support. In Phase 1 the theoretical background and qualitative data from 4 focus groups were combined to define the main components of the intervention. The preliminary intervention was conducted in Phase 2. This was a pilot study conducted in two general practices and amongst 24 patients and 4 peer supporters. Focus groups and semi structured interviews were conducted to collect additional qualitative data to inform the development of the intervention.

**Results:**

The four components of the intervention were identified from the Preclinical Phase and Phase 1. They are: 1. Peer supporters; 2. Peer supporter training; 3. Retention and support for peer supporters; 4.Peer support meetings. The preliminary intervention was implemented in the Phase 2. Findings from this phase allowed further modeling of the intervention, to produce the definitive intervention.

**Conclusion:**

The MRC framework was instrumental in the development of a robust intervention of peer support of type 2 diabetes in primary care.

**Trial registration:**

Current Controlled Trials ISRCTN42541690

## Background

Diabetes is a chronic illness which requires the individual to assume responsibility for their own care with the aim of maintaining glucose and blood pressure levels as close to normal as possible [1]. Maintaining optimal glucose and blood pressure levels reduces the risk of diabetes related complications [2,3]. Treatment of diabetes involves psychological, social and physical adjustments to an individual's lifestyle [1]. This can be confusing and overwhelming for people with diabetes [4]. They have to make a complex range of lifestyle modifications sometimes without necessarily noticing any tangible effects [4]. Emotional and quality of life issues need to be attended to as well as physical issues [4].

**Figure 1:**
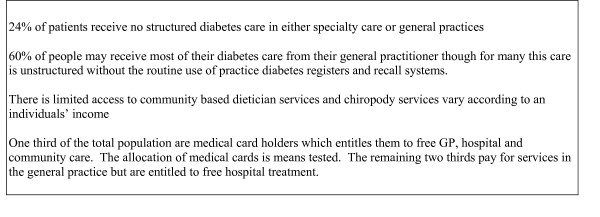
Usual care in the general practice setting for people with type 2 diabetes [31, 32].

Diabetes self management training has traditionally been delivered in a didactic setting with emphasis on imparting knowledge. However this approach has been shown to be ineffective in individual behaviour change and improving metabolic control [5]. In recent times alternatives to the traditional delivery of diabetes care have been investigated, for example, the concept of peer support which emphasises patient rather than professional domination [1]. Peer support could be implemented to complement existing diabetes care. Structured care for people with type 2 diabetes in general practice is not yet well established in the Republic of Ireland [6]. Prevalence of diabetes amongst people over 40 years of age attending 41 general practices in the Republic of Ireland reported a prevalence of type 2 diabetes of 9.2% indicating similar prevalence figures to other European countries [6]. The usual care of patients with type 2 diabetes in the Republic of Ireland is outlined in Figure 1.

**Figure 2:**
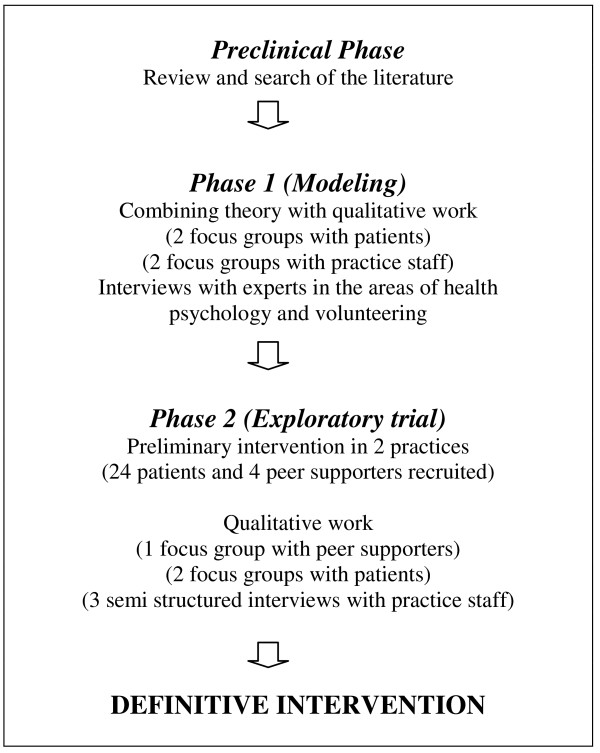
A flowchart of the methodology of the application of the framework.

Testing a complex intervention such as peer support presents a challenge to researchers. Complex health interventions are built up of several components which may include organisational and delivery methods [7,8]. The fact that they involve a number of separate components presents difficulties in isolating the "active ingredient" of the intervention that is effective [8,9]. Therefore it is recommended that a complex intervention for an RCT should be carefully planned and designed [10]. To guide researchers, the UK Medical Research Council (MRC) devised a five phase framework for developing and evaluating RCT's of complex interventions [8]. The framework is comprised of five phases [8]. The Pre-clinical phase involves establishing a theoretical basis to support the intervention. Phase 1, modelling, involves developing an understanding of the intervention and its possible effects. At this point the components of the intervention are delineated. These first two phases are often interrelated. Phase 2, the exploratory trial, is crucial. This is a test of the feasibility of key components of the intervention. Phase 3 is the definitive RCT. Finally long term implementation of the intervention is examined in Phase 4. A flowchart of the methodology of the application of the framework is presented in Figure 2.

This framework has been utilised in a variety of RCT's that have evaluated complex interventions in primary care [7,11-13]. These RCT's examine professionally led interventions for example a behaviour change intervention delivered by primary care practitioners to patients with coronary heart disease [10]. This paper is the first to examine the development of a complex intervention involving peer support.

We describe below, the application of the first three phases of the MRC framework which led to the development of the intervention of peer support in type 2 diabetes based in primary care. The definitive intervention is currently being tested in a cluster RCT, the peer support in diabetes study (Table 1).

**Table 1:** Summary of study

**The peer support in diabetes study**
• **Aims**
To determine whether a peer support programme for patients with type 2 diabetes improves biophysical and psychosocial outcomes and whether it is an acceptable, cost effective intervention in a primary care setting
• **Design**
Cluster randomised controlled trial.
• **Participants**
420 patients with type 2 diabetes recruited from 20 general practices 30 peer supporters, also patients with type 2 diabetes, from 10 intervention general practices.
• **Primary Outcomes**
Blood pressure Total cholesterol HBA1c Well being score [14]

## Aims

### Preclinical phase

The aim of the Preclinical Phase was to review the theoretical basis of peer support and to identify evidence to support the concept.

#### Phase 1

The aim of Phase 1 was to combine the theoretical basis from the Preclinical Phase with qualitative work to define the components of the intervention.

### Phase 2

The aim of Phase 2 was to conduct a pilot study to test the feasibility of the intervention.

## Methods

### Preclinical phase

The Preclinical Phase involved conducting a literature search using CINHAL, Medline and the Cochrane Library. Key words included RCT, diabetes, type 2 diabetes, primary care, community health workers, lay health workers, chronic illness, voluntary workers and peer support workers. The literature retrieved was examined in depth and the concept of peer support was explored. Themes for components of the intervention evolved from reviewing this literature.

#### Phase 1

In Phase 1, the modelling phase, the theoretical basis from the Preclinical Phase was combined firstly with information from interviews with experts in the area of health psychology, diabetes and volunteering and secondly with qualitative data from focus groups with patients with type 2 diabetes and practice staff. Two focus groups (6 patients in each group) were conducted with patients in the two participating general practices. The topic guide included the meaning of the term peer support; the nature of support for people with type 2 diabetes; and an exploration of how peer support differs from professional support. Two focus groups (4 in each group) were conducted with practice staff from the two participating general practices. The topic guide included the definition of peer support; advantages and disadvantages of peer support; and training and support for peer supporters. The focus groups were conducted by a moderator and an observer. Each focus group was taped and the discussions then transcribed and analysed. Descriptive phenomenology was the theoretical framework used for the analysis of the qualitative data. This qualitative research tradition seeks to understand the lived experience of individuals [15]. The combination of information from the Preclinical and this Phase 1 led to the unravelling of four critical components of the preliminary intervention.

#### Phase 2

Phase 2, the exploratory trial/pilot study, involved testing the preliminary intervention. Two general practices were selected. Both are training practices attached to a university post graduate training scheme. One was a small single handed practice and the other a large group practice. Both had a practice nurse and used computerised records. Neither practices had structured diabetes care clinics. Practice staff compiled a register of patients with type 2 diabetes. Twenty two patients and four peer supporters from the two practices were purposefully selected to participate. The peer supporters, who were selected by the GPs, attended two evening training sessions conducted by the research team. The preliminary intervention was delivered in both practices- each peer supporter facilitated three peer group meetings with participating patients over a period of four months. Quantitative data were collected from participants prior to and following the meetings and was analysed using JMP IN statistical package. Qualitative research was also conducted in Phase 2 following the preliminary intervention; two focus groups with five patients each and one focus group with four peer supporters. The topic guide for these focus groups included feedback from the peer group meetings; how peer support differs from support from GPs and practice nurses; and positive and negative aspects of peer support. In addition to these themes the peer supporters were asked about training and ongoing support for peer supporters. The qualitative methodology used was the same as that for phase 1. In addition, three semi-structured interviews with practice staff were conducted following the preliminary intervention. The discussions were based around the logistics of holding the group meetings in the general practices; and recruitment, retention and support for the peer supporters.

## Ethical approval

Ethical approval has been obtained from the Ethics Committee of the Irish College of General Practitioners (Protocol No.: REC0904-11; 01/12/04)

## Results

### Preclinical phase

Theoretical and empirical evidence for peer support was identified in the literature search.

Peer support within the healthcare context is defined as "the provision of emotional, appraisal, and informational assistance by a created social network member who possesses experiential knowledge of a specific behaviour or stressor and similar characteristics as the target population, to address a health-related issue of a potentially or actually stressed focal person" [16]. This definition of peer support falls within the social support model, that is defined as the process through which social relationships might promote health and well-being [17]. Within the social support model, the direct effect model would postulate that peer support could reduce feelings of isolation and loneliness, provide information about access to health services or the benefits of behaviours that positively improve health and well-being and encourage more positive health practices [16].

The logic behind peer support programmes is that peers have a greater understanding of the target population's situation than other naturally embedded social networks [16]. During times of need or in stressful situations individuals often turn to social contacts and relationships for support to supplement the care given by the health services [16].

Members of their own social network may not be able to offer appropriate support for various reasons. For example they may lack experience and knowledge of the stressful life event; they may feel uncomfortable about the issue or are too upset to provide support [18].

Peer support groups provide individuals with a unique support system where they can gain understanding and feel a sense of belonging. As the group evolves attachments are formed and expressions of caring and genuine concern from the group provides emotional support [18].

Peer support was found to be successful in some health care settings. It has improved outcomes in diverse health settings such as maternal child health development[19], neonatal mortality [20,21] and cardiac surgery [22].

Peer support workers also known as lay health workers are defined in a Cochrane review as "any health worker carrying out functions related to health care delivery; trained in some way in the context of the intervention; having no formal professional or paraprofessional certificated or degree tertiary education" (page 1) [23]. Training for peer support workers should incorporate exploration of the skills required to use experiential knowledge and peer's appreciation and understanding of the target group [16]. However Giblin warns against too much specific training, as this may destruct the concept of "peerness" [24]. In addition to peer support benefiting recipients, peer supporters have reported benefits from their role [25-27].

Qualitative research conducted for the Diabetes National Service Framework revealed that people with diabetes felt it would be helpful to meet others in similar circumstances. Peers were viewed as an under-utilised, helpful, source of information and support [28]. However there are no reported randomised controlled trials of peer support in type 2 diabetes. The literature review highlighted the need for a careful consideration of an underlying theoretical framework and the importance of exploratory qualitative work with individuals with type 2 diabetes in the context within which the study was planned.

### Phase 1

In Phase 1, issues raised in the interviews with experts included the identification of social support as a theoretical framework for the study. In addition, experts working in the volunteering sector highlighted the importance of continuing support for the peer supporters to sustain the programme over time.

The patients involved in the exploratory qualitative work expressed enthusiasm for the idea of peer support.

FG1.5 "I thought it would be a good idea for me because from the point of view of the diet it could help me keep me on track. Hearing others ideas and sharing them and so on"

They reported a tendency to turn to peers for advice but felt that a structured support network would be more helpful.

FG2.3 "Very helpful because you are going into a hospital, seeing a doctor, but you are not seeing other people who have it like ourselves"

They had a preference for group rather than individual meetings. Both patients and practice staff felt that peer supporters required specific training that should include the basics of treatment for diabetes and managing a group. However there was a consensus that medical questions from group members should be referred to the GP or practice nurse.

FG7.2 "It is very important for the peer supporters to know their boundaries. They are not doctors"

The work in the Preclinical Phase and in phase 1 led to the identification of four preliminary intervention components:

1. Peer supporters

2. Peer supporter training

3. Retention and support for peer supporters

4. Peer support meetings

#### Phase 2

Phase 2, the exploratory trial/pilot study, involved testing the following preliminary intervention in two general practices:

#### 1. Peer supporters

The GPs and practice nurses in each practice were asked to select two patients with type 2 diabetes who would be suitable for the role of peer supporter. All four peer supporters recruited by the GPs and practice nurses had type 2 diabetes for over a year and were compliant to their treatment regime. Further peer supporter characteristics are presented in Table 2. Findings from the semi structured interviews indicated that the GP's and practice nurses felt they should identify the peer supporters within their own practices.

**Table 2:** Personal characteristic of the patients and peer supporters that participated in the study

	**Patient participants**	**Peer supporters**
Male	13 (59%)	4 (100%)
Mean age (yrs)	66	65
Mean yrs since diagnosis of type 2 diabetes	4	7
Entitled to medical card	14 (64%)	2 (50%)
Smoker	3 (14%)	0 (0%)

#### 2. Peer supporter training

Two evening training sessions were organised for the peer supporters. The content of these sessions included the role of the peer supporter, basics of diabetes, lifestyle and medication issues, communication skills, managing groups, confidentiality, role play and support for the peer supporters. The sessions were interactive and informal. They were given a handbook that covered issues raised in the training session. The focus group with the peer supporters revealed that the peer supporters found the training informative and pitched at the correct level. They valued the handbook and referred to it on several occasions during the course of the exploratory trial.

#### 3. Retention and support for peer supporters

A support system for the peer supporters was implemented. This consisted of the project manager contacting each peer supporter after each group session. This was to allow the peer supporter to debrief and discuss any problems that arose during the course of the meeting. The peer supporters reported that they appreciated this contact.

FG5.6 "Someone out there behind you...Someone behind you saying well how did it go, so you are not left"

#### 4. Peer support meetings

Patients were allocated, by GPs and PNs, to each peer supporter within each practice. Three meetings per group were organised and two groups met in the evening and the other two met during the day. Eighty per cent of patients went to two or three group meetings. Feedback in the focus groups with the peer supporters and patients was positive. Both patients and peer supporters reflected that they enjoyed meeting other people with type 2 diabetes. Exchanging practical information, comparing each others situations, conversing in lay terms and general support amongst the group were identified as particularly positive elements of the group meetings.

FG5.4 "I think there is a common thing here in that the people are not looking for a theoretical understanding of it, you know they don't want to know the Latin. What everybody I think is striving for is kinda practical things"

FG5.4 "the mood was terrific there were delighted to be together they took a lot out of it, there were happy"

Patients and peer supporters agreed that more structure in the group meetings would enhance the peer support experience, for example having a set theme for each meeting. Peer supporters suggested a system of 'frequently asked questions' in order to answer any queries that the group members had identified during a meeting.

FG7.4"after the meeting, somebody should put in their questions into the centre and somebody should answer them and bring it back to the group"

Some peer supporters were anxious to have more professional involvement while others pointed out that this would just reproduce some of the services they currently accessed.

### The definitive intervention

Following the exploratory phase we finalised the study protocol. The definitive intervention is as follows:

#### 1. Peer supporters

Potential peer supporters are identified by GPs and practice nurses in the intervention practices. Peer supporters are recruited and trained at a ratio of approximately one peer supporter to seven/eight patients with type 2 diabetes. They are eligible to be trained if they meet the inclusion criteria outlined in Table 3.

**Table 3:** Summary of the development of the intervention

**INTERVENTION COMPONENT**	**PRECLINICAL**	**PHASE 1**	**PHASE 2**	**DEFINITIVE INTERVENTION**
**Peer supporters**	No formal professional training	To be selected by GPs and PNs	4 peer supporters identified by GPs and PNs	Inclusion criteria: • Identified by GPs and PNs • Have type 2 diabetes for 1 year min • Adherent to diabetes regieme • Understand concept of confidentiality • Liaise with PN/GP if unanticipated problems
		Inclusion/exclusion criteria considered		
**Peer support training**	Non specific training	2 training sessions	2 training sessions- interactive	• 2 training sessions conducted by PN and GP • Conducted locally • Training sessions focused on materials to be used at group meetings • Resource pack/handbook
		Content: basics of diabetes, lifestyle and medication issues, communication skills	Peer supporters handbook	
**Retention and support for peer supporters**		Support for peer supporters vital	Project manager contacted peer supporters following each meeting	Structures in place to ensure retention of peer supporters: • Feasible time commitment to the project • Outline of responsibilities/peer support policy • Adequate training • Resource pack • Contact details and explicit support from the project team and GP/practice nurse • Telephone call from project manager following each session • Annual social event/education session • Travel and related expenses
		Volunteer (no formal payment)	Support from each other at training sessions and focus group following intervention	
**Peer meetings**		7 patients per group	Duration 1–1.5 hours	• 9 peer support meetings per group in 2 years of intervention, held in general practice • 7 patients per group • 10 minute structured component for beginning of each meeting • Any unanswered questions (FAQ) feedback to research team at the end of each session and answers discussed at next session
		3 meetings	Meeting held in general practice	
			Frequently asked questions (FAQ)	

#### 2. Peer supporter training

The peer supporters attend two evening training sessions, which are conducted by a GP and nurse on the research team. Topics covered in Session 1 included: introduction to the project; role of the peer supporter; basics of type 2 diabetes and complications of type 2 diabetes. Session 2 covered the following topics: lifestyle and medication issues; communication skills and working with groups; dealing with difficult group members; role play and confidentiality.

The two sessions focus on the materials to be used during the group meetings (described below) and peer supporters receive a resource pack with a manual and resource material to support these training sessions.

#### 3. Retention and support of peer supporters

Retention of peer supporters is crucial to the study. Structures are in place to ensure peer support workers are supported in the role (See Table 3)

#### 4. Peer support meetings

Peer support meetings are held in the general practice premises at a convenient time for practice staff, peer supporters and participants. The intervention consists of nine peer support meetings held over two years; at month 1, month 2 and every 3 months thereafter. There is a defined ten to fifteen minute structured component for each meeting available to the peer supporters (see Table 4 for a summary of the meeting content). At the end of each meeting there is general discussion and the group identifies and records any questions regarding the meeting focus. These are fed back to the research team who compile written answers based on the feedback from all groups, which are presented and discussed at the start of the next meeting.

**Table 4:** Summary of content of meetings

***SESSION 1- INTRODUCTION***	***SESSION 2- HEART AND VASCULAR DISEASE***
• Introduction to each other • What is peer support? • Ground rules • Discussion on course content (9 sessions) • Video/DVD 15 mins • Entitlements in diabetes • Identifying a substitute peer supporter • Contact details for the group	• Why is it so important? • How you can reduce your risk of heart disease and other vascular complication ◦ Hypothetical individual and what they would advise them to do Questions relating to heart disease including blood pressure and cholesterol medication and taking tablets

***SESSION 3- BLOOD SUGAR LEVELS***	***SESSION 4- HEALTHY EATING***
• Information on hypo/hyperglycaemia • Blood sugar testing Questions on blood sugar levels What to do when you are sick	Discussion of healthy 'eating plate' • Laminated picture of the 'healthy plate' Healthy eating quiz and discussion of answers Questions on healthy eating in diabetes

***SESSION 5- MEDICATION***	***SESSION 6- EXERCISE***
• Control of type 2 diabetes ◦Diet ◦Tablets ◦Insulin Questions regarding medication including side effects	• Importance of exercise • Use of a pedometer ◦each person will be given a pedometer Questions about exercise Maybe arrange a walk in locality

***SESSION 7- FOOT CARE***	***SESSION 8-EYE AND KIDNEY COMPLICATIONS***
• Why foot care matters in diabetes • Discussion on how to check feet ◦Laminated sheet to cover all aspects of foot care Questions relating to the feet Information on local chiropody services	• What happens to the eyes and kidneys in diabetes • Importance of good blood pressure and blood sugar control in order to prevent complications Questions relating to eye and kidney disease

***SESSION 9- LIVING WITH DIABETES***	
This is intended to be a relatively open session in which the group can discuss any remaining concerns and consider whether they would like to continue to meet Importance of follow up data collection	

It became evident to the research team during the Preclinical Phase and Phase 2 that monitoring the delivery of the intervention was crucial. We therefore decided to include a process evaluation and an assessment of treatment fidelity of the definitive intervention. The process evaluation will map the actual implementation of the intervention. Data from peer supporter log diaries of each meeting and the project manager's record of contact with the peer supporters will be recorded and analysed.

The assessment of treatment fidelity will monitor the reliability and validity of the intervention. The Bellg framework will be used. It consists of five treatment fidelity strategies: Treatment design, Training procedures, Delivery of treatment, Receipt of treatment and Enactment of treatment skills [29].

## Discussion

### Summary

Designing complex interventions that are pragmatic enough to be applied to real life situations is challenging [10]. We found the MRC framework very useful in guiding the design and the preliminary testing of the intervention of peer support in type 2 diabetes. The Preclinical Phase explored the existing evidence on the topic of peer support. In Phase 1 the utility of qualitative methods as specified in the MRC framework, and meetings with experts in the field, was invaluable for the early development of the intervention. The preliminary intervention for the proposed RCT was tested in the pilot study in Phase 2. This allowed us to observe the logistics of introducing the preliminary intervention into the primary care setting.

### Methodological issues

After considering several theoretical models and discussing this issue with experts in health psychology and voluntary organisations we selected social support as a theoretical framework for the study. This led to the reassessment of the study outcomes and, in addition to the biophysical outcomes, we added the psychosocial outcomes of wellbeing, self care, self efficacy and social support.

Best practice in randomisation is to randomise following baseline data collection. This avoids introducing bias in terms of patient recruitment and data collection if control practices become demotivated during the baseline data collection phase. Following the exploratory work in phase two, consultation with members of the research team highlighted difficulties with this approach. In order to facilitate the purposive recruitment of peer supporters from the patient register in intervention general practices prior to random selection of patients, it was decided that practices would have to be randomised prior to baseline data collection and randomisation of patients and the beginning of the intervention.

The MRC framework emphasises the importance of monitoring the delivery of the RCT intervention [8]. The review of the literature on conducting randomised controlled trials in the Preclinical Phase and the pilot study in phase two led to our decision to include an assessment of treatment fidelity and a process evaluation in the study protocol. This will allow for the monitoring of the process of implementation of the intervention and also assess the validity and reliability of the intervention. The incorporation of these elements will add depth to our understanding of the final results of the randomised controlled trial. For example, we will be in a position to address any potential questions such as whether the intervention was experienced as intended by the participating intervention patients. In addition, we will be able to consider the relative effectiveness of the intervention in relation to the extent of exposure to peer support. This process will also facilitate reproducibility of the intervention if the trial finds that it is effective as there will be a clear and detailed description of the intervention as it occurred in practice settings.

### Intervention issues

The qualitative work in Phase 1 and Phase 2 allowed us to identify details of the intervention components that needed further development. In particular the structure of the group sessions and support for peer supporters was developed further. The idea of having a focus to each session and a system of frequently asked questions came from the patients and peer supporters and was incorporated into the definitive intervention. A guide for each session was devised. This guide is designed to be flexible and does not have to be strictly adhered to, so as not to destroy the concept of peer led meetings. Unlike the peer led educational interventions such as the Chronic Disease Self-Management Programme (CDSMP)[30] devised by Kate Lorig the intervention in this study focused more on social support than education. There is a clear need to distinguish between interventions that are genuinely peer led compared to professionally led support or educational interventions. As some of the peer supporters emphasised, professionally led interventions would just duplicate some of the services that they currently access.

Consultation with a volunteering expert led to further development of support mechanisms for the peer supporters. The support given in the pilot study, which involved telephone contact after meetings was identified as crucial by the peer supporters and so was developed further for the definitive intervention. We also plan to hold an annual social meeting to facilitate communication between peer supporters from difference practices. The travel allowance for peer supporters has also been modified so that it is given in stages throughout the intervention.

## Conclusion

The MRC framework was instrumental in the development of a robust intervention of peer support in type 2 diabetes in primary care. The intervention of peer support was considered in depth incorporating an analysis of current literature, qualitative work with those who would be both experiencing, delivering and administering the peer support system and finally an analysis of how the intervention would run in the pilot study. It enabled a clear and detailed understanding of the components of the intervention and how each should be documented and tested during the definitive study. The effectiveness of this intervention is now being tested in a cluster randomised controlled trial involving twenty general practices and 420 patients with type 2 diabetes.

## Competing interests

The author(s) declare that they have no competing interests.

## Authors' contributions

All authors reviewed and approved the final version of this manuscript. GP, SMS, TOD and DW designed the study, prepared the protocol and participated in writing this paper. GP conceptualized and drafted the paper, and conducted the data collection. FOK prepared the protocol and participated in writing the paper.

## Pre-publication history

The pre-publication history for this paper can be accessed here:


